# Demonstrative Midwifery—Trial for Libel

**Published:** 1850-07

**Authors:** 


### EDITORIAL DEPARTMENT.

*Demonstrative Midwifery—Trial for Libel.*—A libel suit before the Court of Oyer and Terminer, now in session in this city, has just been concluded, which, from the parties interested being members of the medical profession, and the libelous publication relating to the subject of demonstrative midwifery, it is proper, as a medical journalist, that we should notice. Our readers are already acquainted with the fact that the successful effort to associate demonstrative, with didactic instruction in midwifery, in the Medical Institution of Buffalo, aroused no small degree of opposition among a portion of the medical profession of this city. The letter addressed to the editor of this Journal, and inserted in the number for March, 1850, is sufficient evidence of this fact. In addition to this, considerable excitement among a portion of the community on the subject was produced. How the latter became developed, and the connection subsisting between the opposition in the ranks of the profession, and the excitement existing, to some extent, in the public mind, we will not now discuss, but confine ourselves to the statement of a few facts. The first publication relative to the subject of demonstrative midwifery, was the article contained in the number of this Journal for February, 1850. This article embraced some resolutions adopted by the candidates for graduation attending lectures at the Medical College, expressing their gratitude for the advantages of clinical and demonstrative instruction afforded at the College; together with a letter from Prof. White in acknowledgment of the resolutions, and a few editorial remarks, by way of preface, in which the opinion was expressed, that the plan of teaching midwifery which had been adopted in the Medical College of Buffalo would meet the cordial approbation of the medical profession. We refer to this article to correct some misapprehensions, or, rather, misstatements, and because its propriety has been called in question. It has been said that the adoption of the resolutions was procured through the agency of the Professor of Obstetrics. This was positively denied, under oath, by those who were appointed a committee to draft the resolutions. It has also been said that they were inserted in this Journal at the solicitation of the individual just referred to. This we aver to be false. They were handed to us by the authorized agent of the graduating class, and inserted without consultation with any person on the subject. To say this, is due to the Prof. of Obstetrics, and to ourselves. The resolutions and the letter were published, because the graduating class requested their publication, and we conceive, not only that they were intrinsically entitled to insertion, but that it would have been censurable to have denied them a place in the Journal. Nor are we persuaded of any impropriety in expressing an editorial opinion that teaching midwifery by demonstration would meet with the cordial approbation of the medical profession, albeit it called forth the extraordinary letter signed by seventeen physicians of Buffalo! We leave it for our readers to decide whether a degree of presumption was displayed in the utterance of such an opinion, greater than in seventeen physicians of Buffalo assuming to denounce it in behalf of the whole profession of the United States! That our opinion was correct, is much more demonstrable now, than then, and we reiterate the opinion, and are prepared to offer pretty decisive evidence of its correctness—but this we leave for the present.

The next publication on the subject of Demonstrative Midwifery, was an editorial article in the Buffalo Daily Commercial Advertiser. Much has been said, in this city, respecting the impolicy and impropriety of the publication of this article, on the ground that its effect was to open the subject before the community, and invite popular discussion of it. We think, with others, that the subject is not a proper one for public discussion, although we differ with many as to the probable result of a fair exposition of its merits, and an appeal to public sentiment. But the truth is, the subject had been already brought before the public, not, it is true, by any formal, fair appeal through the press, but by means of rumor and gossip, with all the distortions, exaggerations, and animadversions which might be expected under such circumstances. We shall not stop to inquire by whom, and for what purposes, the subject thus became the theme of public conversation at the corners of the streets, in taverns, and the sick chamber. This we leave for the imagination of our readers, being resolved to say nothing in our journal, or elsewhere, which shall be inconsistent with the utmost allowance of charity. The article in the Commercial Advertiser was written, avowedly, to quiet public excitement, by assuring the numerous readers of that most respectable print, that the Editor had examined into the facts, and that there was no occasion for any popular dissatisfaction with the demonstration at the College. Should the correctness of this statement be doubted by any one, we will copy the article referred to, which speaks for itself on this point. The publication for which an indictment for libel was obtained, purported to be a rejoinder to the editorial article just referred to. The libelous publication appeared, as a communication, in the Buffalo Daily Courier of Feb. 22d, 1850. The author of this communication declares that he claims the right to reply to the writer in the Commercial Advertiser, who "has attempted to defend a gross outrage upon public decency;" and in the next paragraph he asserts what has just been stated with regard to the state of public opinion prior to the publication in the Commercial. We quote the language, italicising the passage relating to this point. He says, "I speak of the article in the Commercial of Tuesday, which refers to the recent 'clinical' exhibition at the 'University of Buffalo, Medical Department,' *an article which was evidently intended to foil public opinion, already setting strongly against the perpetrators of the indecency*, and through the respectability of the print in which it appeared to give that sentiment another direction."

The matter charged as libelous in the remainder of the communication is contained in the following quotations, the italics being ours:—"An *open* demonstration of obstetrical practice has been made before a class of medical students. *The demonstration consumed nearly or quite eight hours, during a part, at least, of which, the professor of that branch of medical instruction was present. Delicacy forbids me to touch upon the manner in which those hours were passed—suffice it to say that the tedium was relieved by such methods as a congregation of boys would know well how to employ.* Thus stand the facts." * * * "*No school on the face of the earth ever tolerated a like exhibition, save the 'Medical Department of the University of Buffalo.'*" * * * "The patient was a woman in humble circumstances, whose poverty, perhaps, overruled her natural modesty. What mattered it, then, if a score of *scarcely adolescent youths satisfied their meretricious curiosity at her expense?* The Professor had enjoyed his 'clinique,' and his class their *salacious stare*, and under the specious plea of scientific advancement a precedent had been set for *outrage indiscriminate*."

For the publication of the foregoing, *Horatio N. Loomis M. D.* was indicted by the Grand Jury of this County in April last, and the trial, which was adjourned in May, came on during the June term of the Circuit Court.

It appeared on the trial, that the communication complained of as libelous, was not written by Dr. Loomis, but that his agency in the publication consisted in his procuring it to be set up at the Courier office after it had appeared in the daily edition of the paper, and the type had been distributed, in consequence of which it was inserted in the weekly issue; in his purchasing extra copies, distributing them, reading the article to his patients, and stating that he had been assured of the accuracy of the statements it contained.

The objects of the complainant, Prof. White, in procuring the indictment we understand were, first, to establish to the full satisfaction of the community and the profession, the facts which actually transpired in connection with the demonstration of labor at the College, and, in this way, to vindicate the transaction, the Institution, and himself, from aspersions of which the communication complained of was only a signal instance; and, second, that medical testimony for, and against the utility and propriety of demonstrative teaching might be brought before the ordeal of a legal tribunal. With reference to these objects, as we are informed, stenographical minutes of the trial were taken, and a full report will be published.

A digest of the testimony of those who were present at the demonstration, and the medical opinions elicited at the trial, will probably appear in the next No. of this Journal. Since we have given the statements alleged to be libelous, it is proper to state here that they were not sustained. The reader will perceive, when he reads the evidence, that the demonstration, instead of comprising nearly eight hours, lasted from *two to five minutes*, and that so far from there having been any gratuitous exposure, the genitalia were protected by the hands of the Professor enveloped in napkins, so as to render it doubtful if any part of them could be seen; that the conduct of the class in the lying-in apartment was characterized by the most perfect order and decorum; that so far from this mode of illustration being a novelty, it is practised at Paris, at Berlin, at Giessen, at Prague, at Heidelburg, at Amsterdam, and at Dublin; that instead of being "boys" or "adolescent youths," the graduating class were all, legally, *men*, i. e. at least twenty-one years of age, and that no grounds whatever existed for charging "*meretricious curiosity*," or a "*salacious stare*" upon those for whose improvement the demonstration was instituted.

With respect to the medical testimony, a number of the Physicians of this city were examined, on the part of the prosecution, and defense, some testifying that such demonstrations are proper and useful, and others that they are unnecessary and improper. Some distinguished members of the Profession from abroad were present, viz. Professors Gilman, of New York;

Ackley, of Cleveland; Coventry, of Utica; Lee, of New York; Webster, of Rochester; Dr. Ganson, of Batavia; all of whom, with one exception, were summoned on the part of the prosecution, and all testified to the propriety and utility of demonstrative midwifery.

The trial commenced on Monday, and was not concluded until Friday evening. It was conducted with great ability on both sides. The counsel for the defendant were Hon. N. K. Hall, Henry W. Rogers, Esq., and James O. Putnam, Esq. In the prosecution, Hon. Henry K. Smith was associated with the district attorney, Benj. H. Austin, Esq. The verdict of the Jury was *not guilty*.

Of the justice or injustice of the verdict we have nothing to say. That we have felt an interest in the suit, we do not deny; but this interest has related entirely to the merits of the subject in connection with which the suit originated, and to the motives and ends of those who, with ourselves, are responsible for the credit, or the odium, as the case may be, of the *innovation* which some of our professional brethren profess to consider "offensive to morality and common decency." The consideration of the subject, forced upon us by the extraordinary position taken by a portion of the medical profession of this city, has tended, more and more, to confirm, in our estimation, the importance of uniting with didactic, clinical and demonstrative teaching in obstetrical instruction. In reviewing the medical testimony in the libel case, in a future No., we shall set forth the grounds for this opinion, and, at the same time, consider the reasons offered by those who contend for the *indecency* and *immorality* of demonstrative midwifery.

Objections founded on delicacy and the moral sense, are, so far as our knowledge extends, confined to those of our Buffalo brethren who, at the outset, deemed it incumbent to enter a protest on the subject. We have had some opportunity of comparing views with members of the medical profession in other and different portions of the country, and we have not yet met with any who have taken similar ground. Some have doubted the practicability, or the expediency of the *innovation* at present, but the preponderance of medical opinion we believe to be in favor of its adoption by medical Institutions generally. This we have no doubt will be the case ere many years elapse. As a pretty fair criterion of the prevailing sentiment with the Profession, we may refer to the various medical Journals of the United States, nearly all of which have spoken in favor of demonstrative midwifery, and not one in opposition to it. The remarks by our contemporaries, on this subject, we shall, in a future No., present to our readers. Our brethren who were impelled to repudiate their fractional participation in the imputation involved in the opinion we ventured to express, that the plan of demonstrative teaching in midwifery would meet with the cordial approbation of the medical profession, will probably be surprised at another assertion which we do not hesitate to make, to wit, that the plan will be sustained by the community at large. We do not regard, as we have already said, the subject one which legitimately falls under the cognizance of public opinion, more than other analogous subjects relating to medical education. But were it necessary, or proper, to resort to public sentiment, and this were done with a fair exposition of the objects to be attained, together with a candid consideration of motives and advantages, without any appeal to prejudices, we should have no doubt as to the result. The subject, unfortunately, has been agitated in this community, not in the manner just mentioned, but by communications in the public prints, deemed by a grand jury libelous, by inflammatory handbills distributed from door to door, and in other modes less conspicuous, but more insidious; and, yet, we are by no means certain that, even under these circumstances, the preponderance of public sentiment among those who have investigated facts, is against it. This, of course, is a matter of opinion, which it is our right to entertain and utter freely, as much as others are at liberty to differ therefrom, as some, if not many, will doubtless do.

The verdict of the *People vs. Loomis*, it is obvious, cannot, with any propriety, be claimed as a disapproval of demonstrative midwifery by a jury of citizens. The jury were not called upon to decide on this matter. In fact, the propriety and utility of demonstrative teaching in obstetrics had but a remote connection with the points at issue, and were admitted rather by mutual consent, and the indulgence of the Court, than from any direct relevancy to the case. Disclaiming, as we do, any feelings having reference to individuals, the ends of the trial, in so far as they have any interest for us, are wholly irrespective of the finding of the jury.

The report of the trial will doubtless be read with interest by the medical public. As connected with the introduction of a mode of teaching instituted alone for purposes relating to the progress of practical instruction in this country; as involving *medical ethics*, and affording indications of medical sentiment of the present time, the trial will rank among the events which make up the history of medicine, and be cited, perhaps, a century hence, as one of the many curiosities which that history embraces.

At the time the grand jury indicted Dr. Loomis, a bill was also found against John E. Roby, Editor of a weekly paper styled the *Christian Advocate*, published in this city, for a libelous publication on the same subject. This cause has not yet come to trial.

